# Exploring the Potential of Generative Adversarial Networks for Synthesizing Radiological Images of the Spine to be Used in *In Silico* Trials

**DOI:** 10.3389/fbioe.2018.00053

**Published:** 2018-05-03

**Authors:** Fabio Galbusera, Frank Niemeyer, Maike Seyfried, Tito Bassani, Gloria Casaroli, Annette Kienle, Hans-Joachim Wilke

**Affiliations:** ^1^IRCCS Istituto Ortopedico Galeazzi, Milan, Italy; ^2^Center for Trauma Research Ulm, Institute of Orthopedic Research and Biomechanics, Ulm University, Ulm, Germany; ^3^SpineServ GmbH & Co. KG, Ulm, Germany

**Keywords:** generative models, spine imaging, synthetic image, *in silico* trial, generative adversarial networks, synthetic spine radiology

## Abstract

*In silico* trials recently emerged as a disruptive technology, which may reduce the costs related to the development and marketing approval of novel medical technologies, as well as shortening their time-to-market. In these trials, virtual patients are recruited from a large database and their response to the therapy, such as the implantation of a medical device, is simulated by means of numerical models. In this work, we propose the use of generative adversarial networks to produce synthetic radiological images to be used in *in silico* trials. The generative models produced credible synthetic sagittal X-rays of the lumbar spine based on a simple sketch, and were able to generate sagittal radiological images of the trunk using coronal projections as inputs, and vice versa. Although numerous inaccuracies in the anatomical details may still allow distinguishing synthetic and real images in the majority of cases, the present work showed that generative models are a feasible solution for creating synthetic imaging data to be used in *in silico* trials of novel medical devices.

## Introduction

The introduction of an innovative medical technology such as an implantable device in the market is a long and expensive process, which is strictly regulated by the competent authorities, with the aim of ensuring safety and efficacy of the product. Depending on the specific regulations, a full campaign of pre-clinical tests may be required prior to the first use of the device in human subjects. Typically, this pre-clinical testing stage includes biomechanical testing in human or animal specimens and implantation in a suitable animal model for *in vivo* testing (Wilke et al., 1998). Subsequently, testing in a human clinical trial is performed, following a sequence of phases involving a growing number of patients and increasing follow-up periods. These required series of activities results in an average cost in the order of millions of dollars prior to the marketing approval of the medical device and a time-to-market of several years (Medical Device Innovation Consortium, 2016).

Recently, *in silico* trials emerged as a disruptive technology, which may reduce the costs related to the development and marketing approval of novel medical technologies, as well as shortening their time-to-market, with an enormous potential impact on both human health and the medical industry (Viceconti et al., 2016). An *in silico* trial has been defined as “the use of individualized computer simulation in the development or regulatory evaluation of a medicinal product, medical device, or medical intervention” (Avicenna Alliance). In practical terms, conducting an *in silico* trial would involve the automated computational simulation of the behavior of the device in a large set of virtual patients, in which specific aspects of the outcome such as the device safety or its biomechanical effect are predicted, and the consequent statistical analysis of these computational results.

Although early examples of *in silico* trials have been presented (Glinka and Polak, 2014; Williams and Mirams, 2015), the use of this form of investigation is not widespread yet, due to the major technical challenges involved (Viceconti et al., 2016). First, methods to simulate numerically the implantation of a medical device in the human body and to predict its outcome in an accurate and valid manner need to be available. Second, a large database of virtual models of patients in which the device should be simulated needs to be generated. The first challenge has been widely confronted in the last decades (Prendergast, 1997), and refined methods to create numerical models based on patient data, typically medical imaging such as X-rays, CTs and MRIs, have been developed and are currently widely employed. The second challenge, on the other hand, emerged more recently and is relatively unexplored (Viceconti et al., 2015), since the vast majority of numerical models were aimed to simulate a single specific patient anatomy and were not intended to be applied to a large population. In fact, large databases of patient data, such as medical images, to be used in future *in silico* trials either do not exist at all or are at least not publicly available. The use of radiological datasets available at hospitals is not an optimal solution to the aim of building simulations in an automated setting, due to the strong heterogeneity and incompleteness of the data as well as ethical concerns related to privacy and security (Häyrinen et al., 2008). Furthermore, the available data may not sufficiently cover the target of the specific *in silico* trial in terms of the characteristics of the pathology and required inclusion and exclusion criteria such as age, sex, comorbidities etc.

Generative models based on deep learning methods are quickly gaining interest as tools to generate synthetic images based on other images or sketches, and already showed promising results in a wide range of applications (Salakhutdinov, 2015; Goodfellow, 2016; Isola et al., 2017). In this work, we propose the use of generative models to create synthetic data to be used in *in silico* trials. The hypothesis of our study is that generative models would allow for the creation of a large number, virtually infinite, of synthetic radiological images, which can be used as the base for an *in silico* trial, in a controlled environment, which allows the designer of the trial to create images perfectly suitable for the intended use. Furthermore, we hypothesize that generative models may be employed to improve the completeness of the data, i.e., to integrate data available from other sources (e.g., from the radiological database of a hospital) whenever the available data is not sufficient for the generation of the computational models.

To test the hypotheses, generative models were used in two distinct scenarios: (1) the generation of synthetic planar X-ray images of the lumbar spine, based on a simple image depicting the outline of the desired anatomy; (2) the creation of sagittal radiographic projections of the trunk and pelvis using the coronal projection as the sole input, and vice versa. For these purposes, generative adversarial networks (GANs) (Goodfellow et al., 2014), which are recently emerging as one of the most promising and successful frameworks for the generation of synthetic imaging data, were used in the present study.

## Materials and methods

### Generative adversarial networks for image-to-image translation

The problem of generating data such as images by means of deep learning methods has been one of the most heavily investigated topics in the field of machine learning in recent years. A description of the technical details of the recent developments is out of the scope of this paper, which is focused on exploring the possible medical applications of GANs rather than on the methodological side. Indeed, a wide technical documentation about GANs as well as other deep learning generative techniques is available elsewhere (Goodfellow et al., 2014; Radford et al., 2015; Zhao et al., 2016).

In simple terms, the use convolutional neural networks to generate realistic images tends to result in blurry outputs, which can be easily detected as fake by the human eye (Isola et al., 2017). The poor outcome is due to the fact that the training of the network requires the definition and optimization of a loss function, i.e., an quantitative measure of the implausibility of the generated output with respect to real images, which is difficult to design in mathematical terms. In early implementations, the employed loss functions used were frequently very specific and application-dependent, thus limiting the applicability of the network to a restricted set of images, or excessively simplified such as the Euclidean distance with respect to the training dataset, which typically results in images lacking sharpness. A brilliant solution to such challenge has been proposed by Goodfellow et al. (2014), who introduced the concept of adversarial networks. In this framework, the generative model, which produces the image is confronted by a discriminative model, which decides if the image is realistic or not, i.e., if the image is coming from the training data or from the generative model. The competition between the two networks leads to an improvement of both the realism of the generated images and the capability of the discriminative model for identifying the implausible images. Blurred images would be easily depicted as fake by the discriminative model and thus discarded. The authors efficaciously described the adversarial nets concept as a team of counterfeiters (= generative network) trying to generate fake currency without being discovered by the police (= discriminative network), which results in improvements of the techniques used by both counterfeiters and police (Goodfellow et al., 2014).

The use of GANs for the image-to-image translation problem, i.e., for generating an image based on another image representing the same scene, has been described in several recent papers. Isola and colleagues introduced a framework in which GANs are used in a conditional setting (Mirza and Osindero, 2014; Isola et al., 2017), which has been widely used for several applications and further investigated due to its generality and simplicity (Huang et al., 2017; Yi et al., 2017; Zhu et al., 2017). In the current study, we employed an implementation of this framework based on Tensorflow, which is publicly available at https://github.com/affinelayer/pix2pix-tensorflow.

### Generation of synthetic planar X-rays from label data

**Figure 1:**
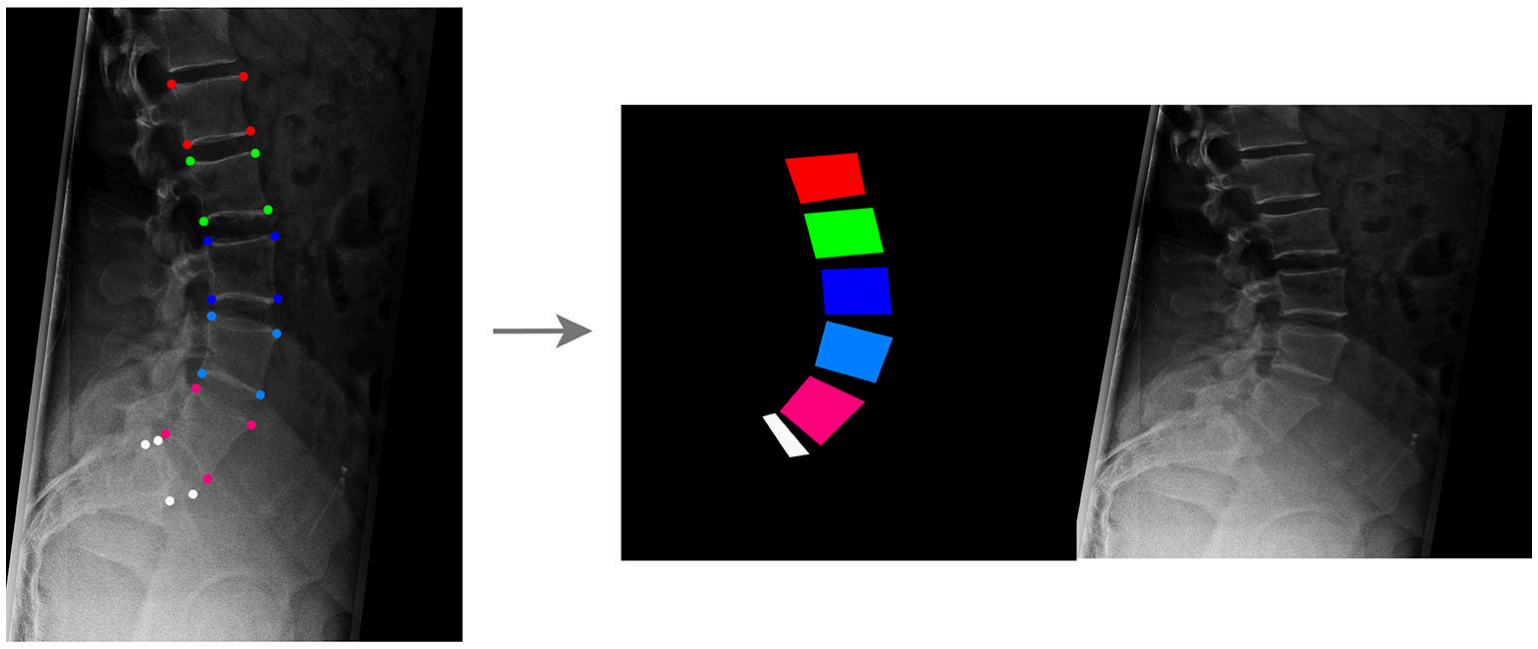
Creation of the training dataset for the GANs aimed to generate synthetic planar X-rays from labels. Vertebral corners, from L1 to L5 and for the upper aspect of the sacrum, are manually identified in each image **(Left)**; based on the coordinates of the points, an image containing the label data on the left and the target radiograph on the right is generated **(Right)**.

To test the potential of GANs in generating realistic X-rays images, a large training dataset has been generated from 1,352 real sagittal radiographs of the lumbar spine. For each image, an operator manually identified the four corners of the vertebral bodies between L1 and L5, as well as four points describing the shape of the upper part of the sacrum including the S1 endplate in a consistent way (Figure 1). If the corner points of one or more lumbar vertebrae or the sacrum were not clearly visible in the radiographic image, as occasionally occurring for example for the sacrum due to the overlapping of the pelvis, the operator did not perform the corner identification for the specific bone.

For each radiographic image, an image having the same size of the corresponding radiograph and depicting vertebrae as colored convex quadrilaterals was generated based on the coordinates of the corner points, using a custom C++ code developed in-house (available at https://sourceforge.net/projects/sketchfromxrays). A fixed color code was used to identify each vertebral level. Both the image containing the labels and the original radiograph were then resized to 256 × 256, and a composite image with size 512 × 256 including the labels on the left side and the resized radiographic image on the right was generated (Figure 1).

**Figure 2:**
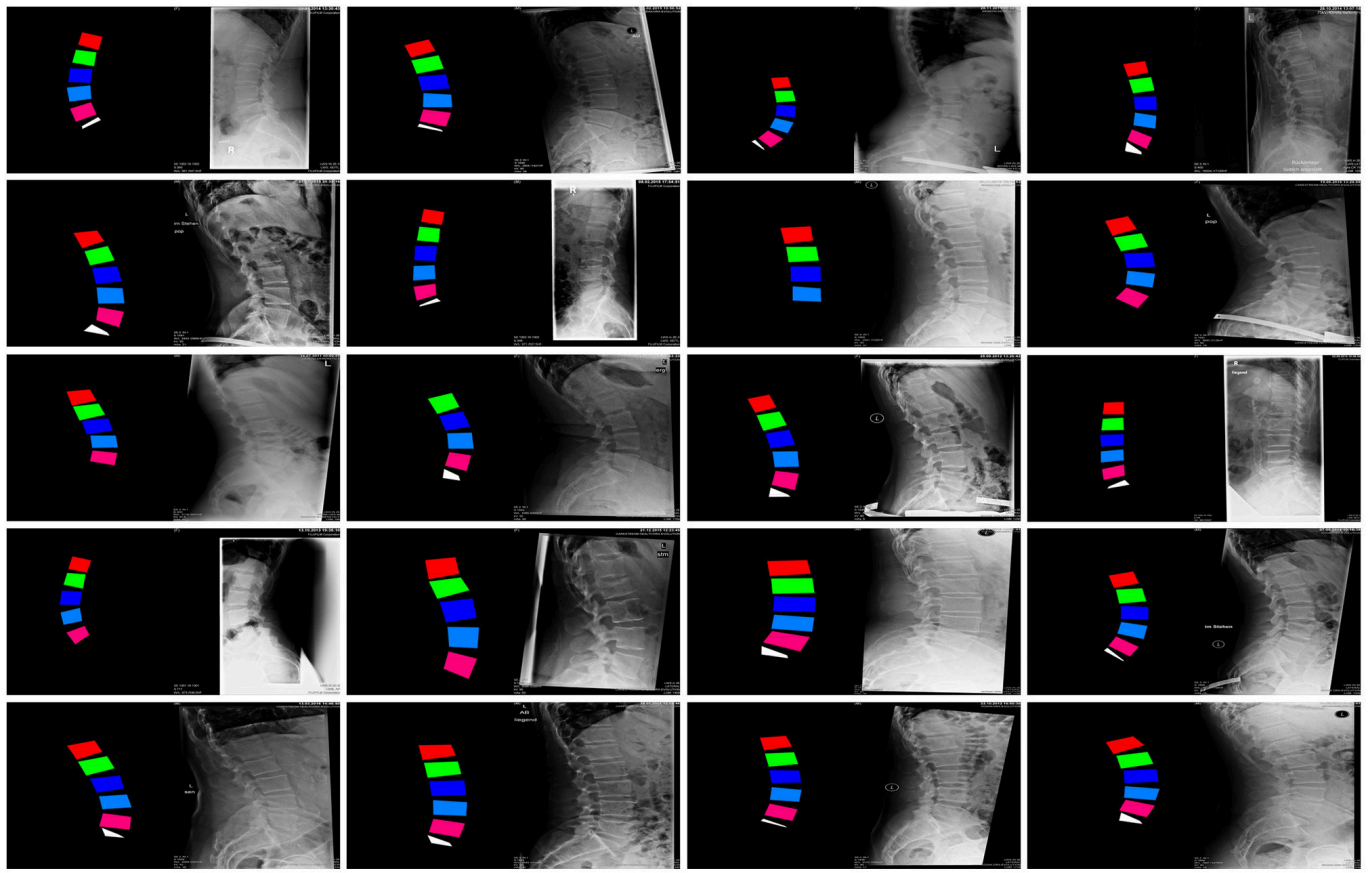
Examples of images in the training dataset used to train the GANs for the generation of synthetic planar X-rays from labels.

Of the collection of 1,352 images, 1,252 were used for training the GANs whereas the remaining 100 constituted the testing dataset (Figure 2).

### Generation of synthetic coronal images from sagittal ones and vice versa

**Figure 3:**
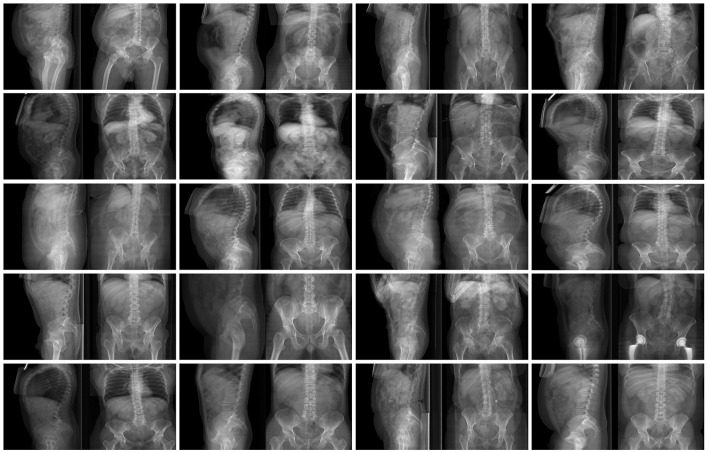
Examples of images in the training dataset used to train the GANs for the conversion from coronal to sagittal X-rays and vice versa.

In order to explore the use of GANs in a more challenging environment in which input and output images have a very different content and appearance, the conversion from sagittal to coronal radiographic projections of the trunk and vice versa has been performed. In order to collect the necessary training data consisting of coupled sagittal and coronal images of the same patients, 1602 CTs have been used to generate simulated projections in the two directions by means of average intensity projection. 72% of the CTs covered the whole trunk, from the shoulders to the pelvis, whereas the rest included only lumbar spine, pelvis and proximal femurs. Similarly to the previous test case, a composite image with size 512 × 256 including the sagittal projection on the left side and the coronal one on the right side was generated for each CT scan (Figure 3). One thousand five hundred two composite images were used for training the GAN, whereas the remaining 100 were employed for testing purposes.

## Results

### Generation of synthetic planar X-rays from label data

**Figure 4:**
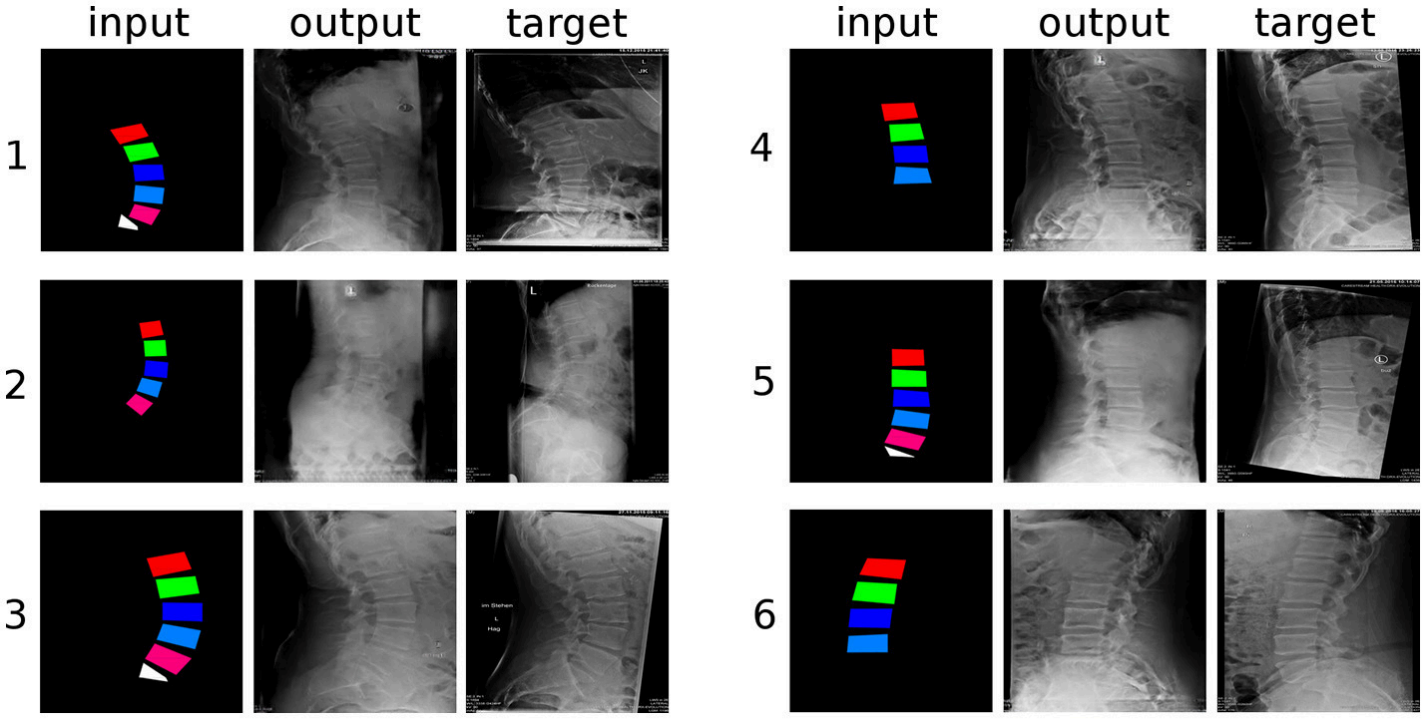
Six randomly selected examples of generated sagittal radiographs of the lumbar spine. “input”: label data provided as input; “output”: image created by the generative model; “target”: ground truth.

The generative framework proved to be able to create convincing synthetic planar X-rays with good success (Figure 4). Based on a colored input image depicting the approximate shape of the lumbar vertebrae and the sacral endplate, the GANs could create realistic representations of lumbar pedicles, facet joints, spinous processes, sacrum, skin, bowel, and ribs.

From a qualitative point of view, the networks had greater difficulties in generating the structures which were not depicted in the input images, such as the spinous processes and the distal part of the sacrum, which turned out rather indistinct and with indefinite contours, with respect to the vertebral bodies, which are directly represented in the inputs. The thoracic vertebrae, some of which are typically visible in lumbar X-rays but about which no information was provided, were also rendered in an approximate manner. It should be noted that these less well defined anatomical structures had a shadowy, indistinct appearance, which nevertheless resulted rather convincing to the human eye. When incomplete data was provided in the input, such as the examples “4” and “6” in Figure 4, which do not include L5 and the sacral endplate, the GANs attempted to render the missing anatomy in the output, with partial success. Interestingly, the networks also replicated some text, which is commonly added to the X-rays and was included in the training data, such as the indication of the side (“L” in Figure 4), the name of the hospital and patient information.

### Conversion from coronal to sagittal X-rays and vice versa

**Figure 5:**
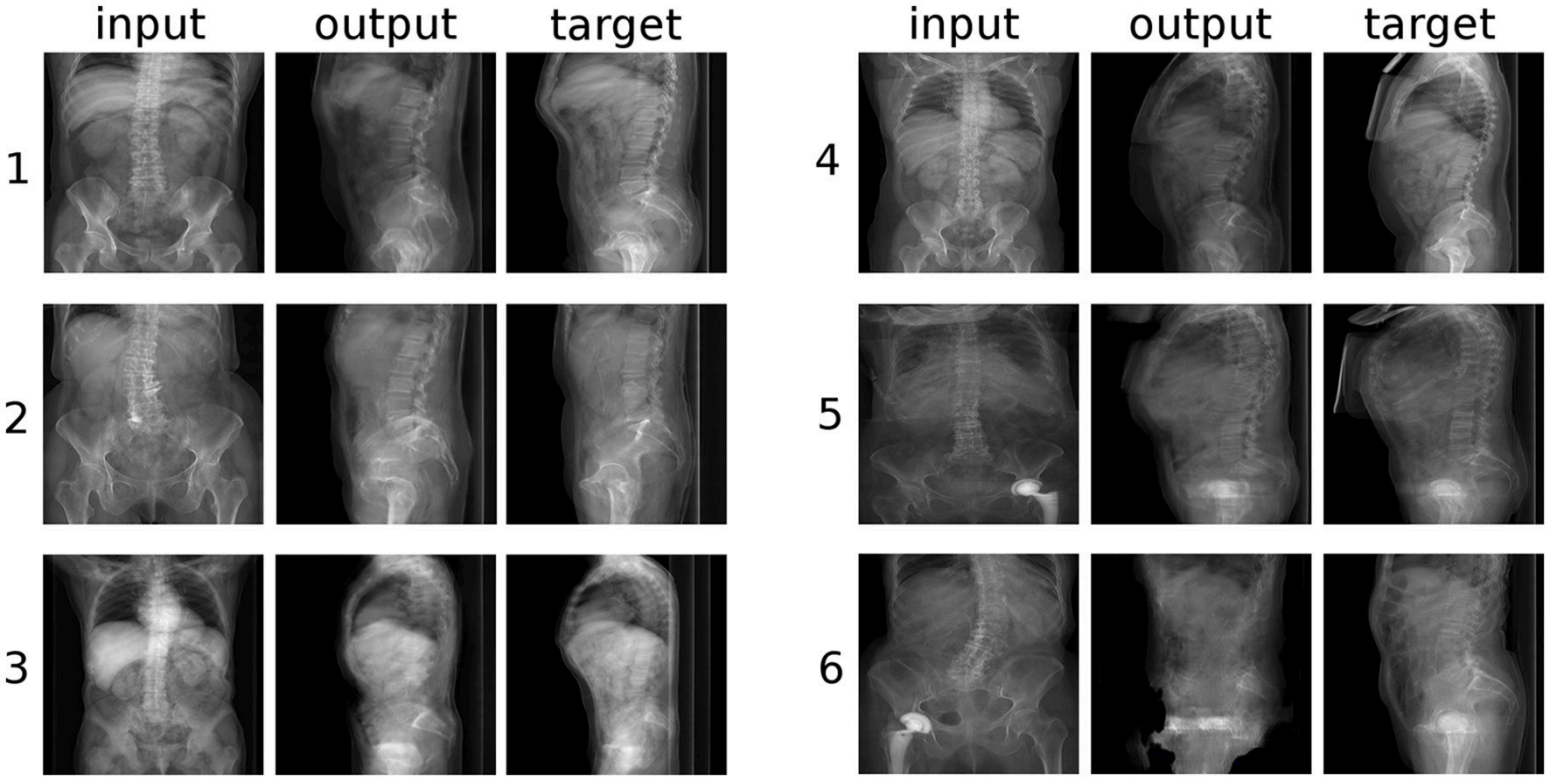
Six randomly selected examples of the conversion from coronal to sagittal radiographic projections of the trunk. “input”: label data provided as input; “output”: image created by the generative model; “target”: ground truth.

**Figure 6:**
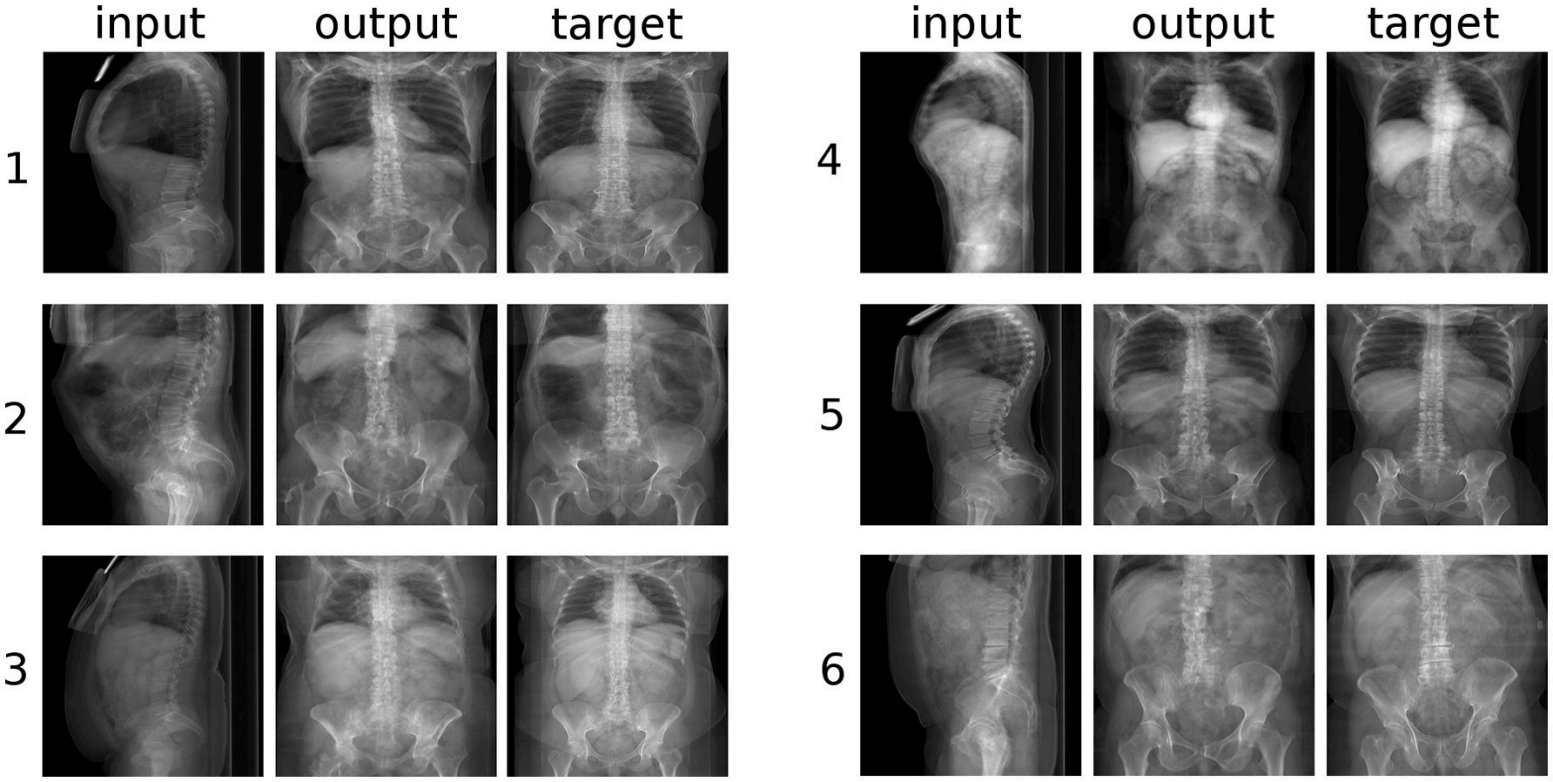
Six randomly selected examples of the conversion from sagittal to coronal radiographic projections of the trunk. “input”: label data provided as input; “output”: image created by the generative model; “target”: ground truth.

At a first glance, the GANs were able to generate credible sagittal X-rays projections of the trunk based only on the coronal projection (Figure 5), and vice versa (Figure 6). The output of the networks included all and only the anatomical structures depicted in the input, and respected the general appearance of the input image. For example, for images taken with a low X-rays dose in which the internal organs are clearly visible, such as example “3” of Figure 5 and example “4” of Figure 6, the corresponding output also showed renderings of the organs with correct sizes, positions and densities. The GANs also produced a remarkable output in terms of the general body size, i.e., distinguishing between patients with high body mass index and large body envelope and thinner subjects. The occasional presence of a hip joint replacement was also generally correctly rendered (see example “5” in Figure 5).

However, a closer inspection of the generated images revealed several anatomical inaccuracies. In the sagittal images (Figure 5), whereas the rendering of the lumbar spine and of the sacrum was mostly realistic, the geometry of the pelvis and of the hip joints was generally rather indistinct. The coronal images (Figure 6) showed in most cases a credible representation of the upper trunk including the shoulders (whenever visible), the thoracic spine and the ribs. Although the generated pelvises were realistic and symmetric, the femurs were inaccurately represented in some cases (e.g., example “2” in Figure 5), especially with respect to the shape of the trochanters. The anteroposterior rendering of the lumbar vertebrae was rather satisfactory in several cases (such as examples “1”, “3,” and “5” in Figure 6) and included pedicles, laminae and spinous processes; in other cases (examples “2” and “6”), the depiction was asymmetrical and generally unrealistic. These inaccuracies are likely related to the composition and size of the training data; the relatively low number of images of the lumbar spine determined a generally lower quality of the outputs regarding lumbar images with respect to those including the whole trunk. In a small number of cases (see example “6” in Figure 5), a clearly implausible image has been generated. Specifically for example “6,” the incorrect output may have been triggered by the concomitant presence of lumbar scoliosis and a hip prosthesis, which was very infrequent in the training data.

## Discussion

The recent developments in generative models, including GANs, are providing novel powerful tools to the scientific community, the possible uses of which are still being explored. In the present work, GANs proved to be able to generate realistic synthetic medical images, which were in several cases challenging to distinguish from real ones even for a human observer. Although the numerous inaccuracies in the anatomical details may still allow for a correct identification of synthetic images in the majority of cases, the potential of GANs, which emerges from analysis of the results shown in Figures 4–6 is evident.

In our work, we employed the conditional GANs framework presented by Isola and coworkers, which has been developed as an application-agnostic tool and that has been tested by the authors in tasks such as the generation of photorealistic images from labels and sketches, and image colorization (Isola et al., 2017). Indeed, current research about generative models, and especially GANs, is now targeting a number of applications such as the colorization of black and white images, the generation of images based on sketches, sparse annotation (Karacan et al., 2016) or even text (Reed et al., 2016), the creation of maps from aerial views and the increase of the image resolution during upscaling (superresolution) (Ledig et al., 2016), with outstanding results highlighting the potential of these methods.

Regarding applications related to medical imaging, the use of generative models seems to be still in its infancy. An example of an early musculoskeletal application is offered in a recent paper by Kadoury and colleagues, who trained a model to predict the curve progression in subjects suffering from adolescent idiopathic scoliosis, and retrospectively validated the results against clinical observations and radiological imaging (Kadoury et al., 2017). Another interesting application is described in a work by Aubert and coworkers, who used convolutional neural networks to remove the shadowing due to the presence of metallic implants in biplanar radiographs, in order to facilitate the three-dimensional reconstruction of the spinal anatomy (Aubert et al., 2017). On the other side, the use of other machine learning methods other than generative models is already rather established in medicine. Concerning musculoskeletal imaging, several papers employed deep learning techniques for classification problems such as the automated grading of disc degeneration and herniation on MRI scans (Koh et al., 2012; Jamaludin et al., 2017), or regression problems such as locating anatomical landmarks in planar X-rays (Galbusera et al., 2016), with promising results, which anticipate a wider exploitation of machine learning in radiology research in the near future.

Based on the results of the present study, the task of generating synthetic images to be used in *in silico* trials seems to be within the reach of generative models. We foresee that future uses of GANs may include the creation of virtual patients to be recruited in *in silico* trials, including a full radiographic dataset, based on a small set of sparse data. A possible example of such application would be the generation of the X-rays and CT scans of a patient suffering from idiopathic scoliosis, based purely on body size, Cobb angles and Lenke classification (Lenke et al., 2003), which may be later employed for the simulation of the surgical treatment. Such a generative framework would allow creating a large group of synthetic patients, which would cover the anatomical and functional variability of the pathology based exactly on the desired inclusion and exclusion criteria of the *in silico* trial. In addition to the generation of complete synthetic patients, GANs may support the management of heterogeneous data, for example if the virtual patients to be recruited in the *in silico* trial are based on real patient data and images, rather than on a fully synthetic dataset. In this case, similarly to the published works in which generative models filled missing regions in pictures (Yeh et al., 2016; Iizuka et al., 2017), the models may help in the standardization of the data structure, by filling gaps in the available data whenever required, thus allowing for an easier automated generation and simulation of the numerical models. Besides these applications strictly related to the creation of *in silico* trials, generative models may prove useful for other related tasks such as automating the segmentation of CT and MRI scans (Diplaros et al., 2007; Sabuncu et al., 2010), extracting the value of anatomical parameters from imaging data, creating virtual multimodal images such as CT/MRI, as well as generating a three-dimensional anatomical model from two-dimensional data (Wu et al., 2016) such as an X-rays projection.

Exploiting generative models to create synthetic patient and radiological data involves, however, overcoming a number of obstacles. First, training the models requires a large dataset of annotated data, in turn necessitating a large database of raw radiological data from real patients, which is not easily accessible by many research institutes (Viceconti et al., 2015). Furthermore, the generation of thousands of sketches or labels from real patient data, such as those shown in Figures 1, 2, may require a substantial amount of manual work if an automated method to perform the task is not readily available. Other foreseen difficulties are associated to the ethical aspects of acquiring, storing and exploiting clinical and imaging data pertaining to real patients, such as privacy and data security (Nunan and Di Domenico, 2013; Schneeweiss, 2014). Due to the potential commercial value of software frameworks aimed to perform *in silico* trials of medical devices, issues related to the marketing exploitation of products deriving from sensitive patient data should also be taken into account (Murdoch and Detsky, 2013).

The present study has some limitations, which should be considered in light of its explorative nature. Only two possible applications of generative models related to *in silico* trials have been tested to date, and both regarded the same anatomical district. As a matter of fact, further tests covering a larger scope of clinical applications need to be performed in order to prove the general validity and usefulness of GANs in the context of *in silico* trials. In addition to the generation of radiological images, the creation of a full dataset of virtual patients and the respective numerical models involves several other key steps, such as for example image segmentation and the automated generation of numerical models based on synthetic patient data. Technical issues can be foreseen in the implementation of specific tasks; for example, virtual multimodal CT/MRI imaging would require the creation of a large training dataset consisting of coupled CTs and MRIs, relative to the same patient and time point, which are practically hard to collect even in the radiological databases of large hospitals. Similarly, generating three dimensional models and finite element meshes based on synthetic data involve numerous technical challenges, which should be considered outside of the scope of the present work but need to be addressed prior to a fully automated implementation of *in silico* trials.

Furthermore, the quality of the outputs of the generative models has been judged qualitatively only by assessing their plausibility, whereas no quantitative evaluation has been performed. Concerning the latter point, it should be noted that assessing the quality of synthetic images in an automated manner is an open and challenging issue, and although a few methodological works about it have been published, a general solution is not currently available (Salimans et al., 2016; Isola et al., 2017). It should be noted that an insufficient quality and plausibility of the generated images may practically prevent their use in *in silico* trials, and therefore constitutes a key issue to be investigated in future studies.

In conclusion, the present work showed that generative models are a feasible solution for the creation of synthetic imaging data to be used in *in silico* trials of novel medical devices. Assuming that *in silico* trials become standard step of the process of developing and bringing to the market a novel implantable device in the near future, generative models have the potential to provide a fundamental contribution in the creation of the cohort of virtual patients to be recruited in the simulated trial.

## Author contributions

FG, FN, AK, and H-JW: conceived the main ideas of the study; AK and H-JW: supervised the project; FG, FN, and TB: developed the various computer programs involved in the project; MS, AK, and GC: assembled the set of annotated images used for training the generative models; FG and AK: were involved in funding acquisition; FG and FN: wrote the draft of the paper. All authors reviewed the manuscript.

### Conflict of interest statement

The authors declare that the research was conducted in the absence of any commercial or financial relationships that could be construed as a potential conflict of interest.
